# Treatment Processes for Microbial Resistance Mitigation: The Technological Contribution to Tackle the Problem of Antibiotic Resistance

**DOI:** 10.3390/ijerph17238866

**Published:** 2020-11-28

**Authors:** Gabriela Bairán, Georgette Rebollar-Pérez, Edith Chávez-Bravo, Eduardo Torres

**Affiliations:** 1 Instituto de Ciencias, Benemérita Universidad Autónoma de Puebla, Puebla 72570, Mexico; gabriela.bairan@alumno.buap.mx; 2 Facultad de Ingeniería Química, Benemérita Universidad Autónoma de Puebla, Puebla 72570, Mexico; georgette.rebollar@correo.buap.mx

**Keywords:** advance oxidation processes, electrochemistry, microbial resistance, ozonation, photocatalysis, treatment technologies

## Abstract

Advances generated in medicine, science, and technology have contributed to a better quality of life in recent years; however, antimicrobial resistance has also benefited from these advances, creating various environmental and health problems. Several determinants may explain the problem of antimicrobial resistance, such as wastewater treatment plants that represent a powerful agent for the promotion of antibiotic-resistant bacteria (ARB) and antibiotic resistance genes (ARG), and are an important factor in mitigating the problem. This article focuses on reviewing current technologies for ARB and ARG removal treatments, which include disinfection, constructed wetlands, advanced oxidation processes (AOP), anaerobic, aerobic, or combined treatments, and nanomaterial-based treatments. Some of these technologies are highly intensive, such as AOP; however, other technologies require long treatment times or high doses of oxidizing agents. From this review, it can be concluded that treatment technologies must be significantly enhanced before the environmental and heath problems associated with antimicrobial resistance can be effectively solved. In either case, it is necessary to achieve total removal of bacteria and genes to avoid the possibility of regrowth given by the favorable environmental conditions at treatment plant facilities.

## 1. Introduction

The advances in medicine, science, and technology since the mid-20th century have helped humans abide by reaching out for a more comfortable and bearable quality of life. However, all these technological and scientific changes have had an impact not only at the humankind scale, but also at the microscopic scale through the so-called antimicrobial resistance (AMR) phenomenon, which is at the origin of a large scale, worrisome, and worldwide issue concerning the health of living organisms and the environmental deterioration. The antimicrobial resistance occurs naturally in the environmental compartments as a response of microorganisms to control microbial growth and colonization of pathogens by developing antimicrobial agents [1]. Its effects started to get noticed with the discovery and use of penicillin when the first resistant bacteria came up from an evolution mechanism to adapt to this antibiotic’s presence. In the 1960s decade, the first antibiotic-resistant strains were reported; such was the case of *S. aureus* to metacycline; ten years later, this bacterium was found to be resistant to ampicillin, and another ten years later, it was also resistant to vancomycin. The extent of resistance evolved quickly with time, in agreement with the increasing use of antibiotics, regardless of when the antibiotics were generated. Plasmid-mediated resistance to colistin was reported since the year 2000, and resistance to ceftriaxone, a third-generation antibiotic, was reported in 2010 [2].

**Figure 1:**
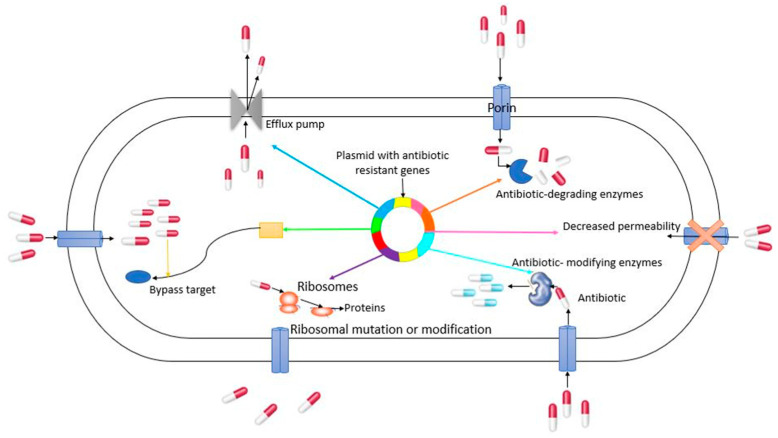
General mechanisms of antimicrobial resistance (Elaborated from references [10] and [11]).

Antimicrobial resistance is the ability of microorganisms to tolerate the effects of antimicrobial therapies. Antimicrobial resistance is determined by the genetic plasticity of bacteria that triggers adaptation responses, allowing genetic mutations, and acquisition of genetic material through horizontal transfer (transformation, transduction, transposition, and conjugation) [3], ensuring the spread of bacterial species [4]. Bacterial genomes harbor different genes encoding antimicrobial resistance (reflecting genotypic resistance). The expression of these genes by the selective pressure of the antibiotic triggers resistance mechanisms such as efflux pumps, cell wall recycling, porins reduction, target protein modification, active expulsion systems, biofilm formation, among others (phenotype resistance) (Figure 1) [5]. However, antimicrobial resistance can be reversible. Resistance reversion can occur at the level of a strain or the level of a population; at the strain level, it happens through mutations or loss of resistance genes that restore the phenotype susceptible to antibiotics, such as modifying the membrane permeability and the activity of the regulators involved in the defense against drugs and related stresses, partially restoring them in a natural environment without antibiotics and reducing the level of resistance. For certain antibiotics, it has been shown that drug resistance decreases over 480 generations during exposure to an antibiotic-free environment, but the loss of resistance can progress slowly [6]. It is caused by a temporary change in the balance between susceptible and resistant strains in a bacterial population at the population level, which may be evident from phenotypic susceptibility [6,7]. This is how different microorganisms become resistant to one or two antibiotics and many others [8]. When antibiotic resistance is developed in microorganisms, medicine becomes inefficient and the infections become more harmful, which increases the cost of hospital treatments and the risk of spread to other people [9].

The myriad of microorganisms resistant to antibiotics represents a present and severe future threat to human health worldwide. The WHO (2017) published a list of “priority pathogens” that are classified as multidrug-resistant, extremely drug-resistant, and methicillin-resistant [12]. These pathogens represent a very high risk to animals or human health, because new antibiotics need to be created to treat infections caused by these bacteria [13,14]. Some microorganisms developed resistance to medicines available since the 2000s decade [15]. The so-called *Enterococcus faecium*, *Staphylococcus aureus*, *Klebsiella pneumoniae*, *Acinetobacter baumannii*, *Pseudomonas aeruginosa*, and Enterobacter species microorganisms (ESKAPE) are pathogens of difficult treatment, because they present a high AMR [16]. These microorganisms migrate between the environmental compartments [17] and are the subject of several research types [18,19]. The severity of the situation derived from AMR is highly concerning based on the estimate published in 2016, which stated that drug-resistant microorganisms caused around 700,000 annual deaths, and these numbers are expected to increase sharply by the year 2050, mostly due to the rate at which antibiotic intake doses have increased [20,21].

The number of studies concerning this problem has been increasing at a fast pace. Since the beginning of the year 2000, AMR started receiving more specific topics to understand this phenomenon better. We conducted a literature review with predefined criteria using the online databases of Scopus and Web of Science to find papers on technologies for mitigation or elimination of ARB and ARGs. The purpose was to provide an updated description to evaluate the technologies and their limitations in the wastewater treatment plant (WWTP) conditions. Some examples of keyword combinations for the research were “mitigation technology”, “microbial resistant bacteria”, “microbial resistant genes”, “advanced oxidation process”, “ozonation”, “electrochemical oxidation”, “photochemistry and photocatalysis”, and “Fenton oxidation”. The results provided approximately 11 thousand articles. A high percentage of these research studies (80%) are grouped in the medical, pharmacology, immunology and microbiology, and the biochemistry fields, which report results on the consequences of antimicrobial resistance in the treatment of bacterial infections, genetical identification of resistant genes, development of new drugs, the mechanisms of microbial resistance of different bacterial species, and the synthesis of new antibiotics. The remaining 20% of the researches are grouped in other important domains, such as agriculture (9.4%), environmental sciences (2.8%), social sciences (0.55%), and engineering sciences (1.4%), showing the complex and multidisciplinary nature of the problem.

**Figure 2:**
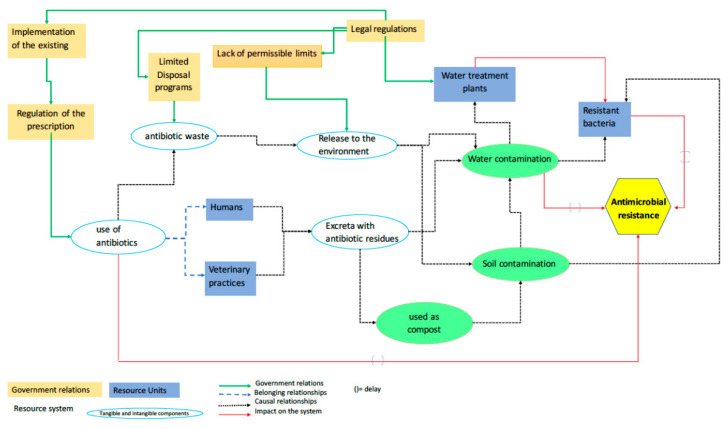
Determinants in the antimicrobial resistance problem.

The interaction among multiple biological, sociological, and cultural determinants have allowed the existence of microbial resistance [22,23,24,25]) (Figure 2). One of the main reasons is the intensive and inappropriate use of antibiotics for human and animal consumption; between the years 2000 and 2015, an increase of 39% was reported in the consumption of antibiotics, not only to treat infections or illnesses in humans and animals, but also to promote animal growth in aquaculture and livestock and poultry farming [26,27]. Due to the intensive use of antibiotics, human and animal excreta contain an excess of antibiotics or their metabolites, which are discharged to the sewer, and are sometimes directly discharged into rivers in places where municipal effluents are not treated [14,28,29,30]. Animal manure used as fertilizer in crops also represents a pollution source in soils and underground and superficial water when infiltration into the soil occurs [31,32].

On the other side, there are currently no legal regulations that define the maximum allowable limits for antibiotics or other resistant-promoting substances emitted to the environment [33], or the regulations have only been adopted in economically large countries. The lack of proper prescriptions, misuse of antibiotics by health professionals, low drug quality, and the inappropriate disposal of expired antibiotics add to the problem’s complexity. The problem’s extent seems to be aggravated because of psychological, cultural, and behavioral practices such as current lifestyles, attitudes, the misconception on the antimicrobial effects of drugs, and public’s knowledge and beliefs about antibiotic resistance [34,35,36,37,38]. Once in the water, antibiotics and ARB multiply and spread their presence to other environmental compartments. As both water and land can be directly affected by industrial, municipal, or agricultural activities, other factors promote selective pressure and enable the amplification, maintenance, and spread of ARB. Moreover, in the wastewater treatment plants (WWTPs), the place where the elimination of chemical and biological pollutants should be eliminated, the environmental conditions such as the presence of metals, pesticides, and abundance of microorganisms promote antimicrobial resistance in a larger number of bacteria or microorganisms by selective pressure [39,40,41]. Table 1 shows a shortlist of several antibiotic-resistant microorganisms that have been found in wastewater treatment plants.

Therefore, it can be observed that antibiotic resistance is a problem of several dimensions in which the interaction of the different elements could give place to new determinants or factors non-existing before. A solid comprehension of human activities’ interaction with animals and the surrounding environment will help develop viable and sustainable solutions against the environmental emergency derived from the propagation and persistence of ARB and their genes. Considering the complexity of the problem, whether wastewater treatment contributes or not to the mitigation of antibiotic resistance is still a gap in the knowledge [62,63]. The WWTPs represent sites for amplification of ARB and ARGs and a valuable factor to mitigate the problem. Effluents from these facilities are usually used for several socioeconomic activities, promoting the problem’s distribution and amplification. Therefore, proper treatment of the effluents could serve as one appropriate step in diminishing ARB and ARGs’ spread. This review focuses on the description of current technologies understudy to elucidate their potential as a treatment process for eliminating ARB and ARGs; one purpose of the contribution is to highlight the limitation so far reported that limits the implementation of technologies in field conditions found in WWTPs.

## 2. Technologies to Abate Microbial Resistance

Currently, there are already published several reviews that summarize the treatment strategies comprehensively to combat antibiotic resistance in WWTPs, which represent one of the major places where antibiotic resistance evolves [13,14,30,64,65]. To reduce the environmental and health impact of ARB and ARGs, it is vital to understand the efficiency and mechanism of the treatment technologies and the limitations for their eventual application. Removal pathways include adsorption, biodegradation, disinfection, and filtration using small pore sized membranes. Other pathways, such as hydrolysis, photolysis, and volatilization, also contribute to removal depending on antibiotic properties [66]. These removal pathways can be observed in anaerobic/aerobic tanks in wastewater treatment plants, constructed wetlands, and advanced oxidation processes, which can be categorized as illustrated in Table 2.

Table 3 shows examples of technologies to abate microbial resistance; the principal results of the references in this table are discussed in the following section.

### 2.1. Anaerobic, Aerobic, or Combined Treatments

Biosorption and biodegradation are considered the principal mechanisms for antibiotic removal, antibiotic-resistant bacteria, and antibiotic-resistant genes in biological processes. Better performance is reached when anaerobic/aerobic processes are coupled in a wastewater treatment process [13]. As expected, the reduction of ARGs is strongly correlated to the digesters’ operating conditions, the season of the year, and the type of ARGs targeted [30]. Other studies have shown that thermophilic anaerobic digestion, in a temperature range of 40–60 °C increases the removal of several ARGs by up to 89% for macrolide resistance genes [87,88,89]. The sludge retention time and pH adjustment also influence ARG removal. More considerable reductions have been shown with long sludge retention times, between 10 and 20 days for genes such as sulI, sulII, tetC, tetG, and tetX and pH adjusted to alkaline conditions, between 9–11 [88]. In principle, alkaline pH levels block ARG dissemination by limiting the number of transferable plasmids and their transformation efficiency [90]. However, not all ARGs are susceptible to being reduced during anaerobic treatments. According to Aydin et al. [91], ARGs from erythromycin and tetracycline increased during anaerobic digestion. Researchers have found that microwave, thermal hydrolysis, and ozone could be coupled to anaerobic digestion for better results in these cases. Microwaves damage the cell membrane leading to disruption in DNA. During thermal hydrolysis, sludge bacteria are sterilized, and cell walls are destroyed, leading to DNA reduction. Finally, the ozone’s nonselective oxidation could be useful, if ozone can penetrate the cytoplasm to achieve gene reduction [30]. Aerobic digestion has been less studied concerning the removal of ARGs. This treatment method seems highly related to hydraulic retention times, the reactor design, and ARG type. For instance, genes such as ermB, sulI, tetA, and tetW have been reduced by up to 85% under aerobic digestion, at a temperature of 20 °C and a 13-day hydraulic residence time [71]. On the contrary, other studies have shown that genes such as tetA, tetX, sulI, intI1, and 16S rRNA have been proven to increase under sludge aerobic digestion [67]. Further studies are needed to clarify this behavior [30].

Concerning antibiotic removal, biological systems have proved to be efficient to a certain extent [92,93], although concentrations within the ng/L range are still detected in the effluents of biological reactors [94,95,96]. Antibiotics such as sulfonamides, tetracyclines, and macrolides have been removed in aerobic, anaerobic, and combined systems [97]. For instance, the elimination rates of five quinolones, five sulfonamides, and four tetracyclines by three sewage treatment plants in Xinjiang ranged from 17% to 100%. The WWTP with the combined technology consisting of anaerobic/anoxic/aerobic step combined with membrane bio-reactor removed the antibiotics more efficiently than the combined technology of anaerobic/anoxic/oxic coupled to the oxidation ditch step [98]. In another report, activated sludge, anaerobic digestion, and conventional membrane biological reactor removed up to 98% of sulfonamides, and 88.9% of tetracyclines from swine wastewater [99]. A high percentage of antibiotic removal is due to adsorption in activated sludges, which must be inactivated in the following process to remove all the antibiotics completely.

Membrane bioreactors (MBRs) are process units that use the principles of mass separation based on molecular size or compound affinity with a membrane of controlled and fine pore size. The membrane acts as a barrier that removes undesired compounds from liquid or gaseous mixtures in an advanced filtration process. Membrane bioreactors can operate in aerobic or anaerobic modes and can be installed in water treatment facilities combined with other processes, such as slurry tanks, or after secondary treatments in wastewater treatment plants, as previously mentioned. Membrane bioreactors have been recently used to remove ARB and ARGs efficiently. For instance, Kappell et al. (2018) [100] reported that a ceramic membrane (0.05-μm pore size) coupled externally to an anaerobic fluidized bed reactor could achieve a 3.3–3.6 log reduction of erm(B), tet(O), and sul1 as well as the horizontal gene transfer determinate intI1. Wang et al. (2020) [101] evaluated the removal of antibiotics (ampicillin, erythromycin, tetracycline, kanamycin, and ciprofloxacin), ARB (*Aeromonas*, *Escherichia*, *Klebsiella*, and *Bacteroides*), and ARGs (ermB, tetO, tetW, and intI1) in five full-scale MBRs (membrane pore size < 0.4 µm). The results showed that the MBRs could achieve high removals of nearly 94% for individual ARB, up to 77.5% degradation of total antibiotics, and three to four orders of magnitude reduction of ARGs. The authors observed the long retention times and the high biomass retention. The main processes behind the high removals of emerging contaminants with MBRs are biodegradation, bioadsorption, and membrane filtration.

### 2.2. Constructed Wetlands

**Figure 3:**
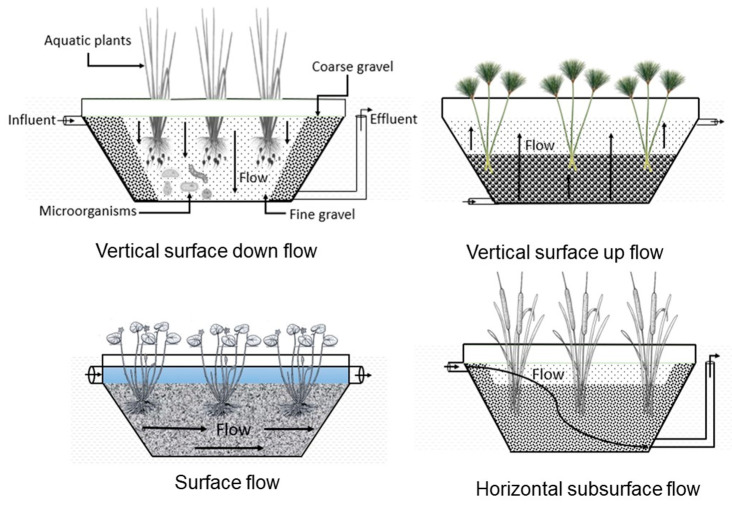
Scheme for flow configuration, type of support, and type of macrophyte in the constructed wetlands.

Constructed wetlands (CWs) are designed and constructed to simulate natural processes that purify water. They have been used to treat domestic, cattle, and municipal wastewater [102,103,104]. These systems were demonstrated to possess the ability to reduce biochemical oxygen demand (BOD), chemical oxygen demand (COD), nitrogen and phosphorus, as well as emerging contaminants of different origin [105,106]. CWs can be operated under different flow configurations, with different macrophytes and different solid supports, and it is thus possible to design specific configurations applicable to remove certain pollutants from effluents [107,108] (Figure 3).

CWs can also represent an alternative strategy to reduce or remove antibiotics and ARB and ARGs in wastewater [109,110]. These systems exhibit a wide range of efficiencies (from 59 to almost 99%) depending on the flow configuration, plant species, flow types, and season [80,111]. The CWs promote persistent micropollutants adsorption from the support or substrate [112,113] and plant uptake [113] as mechanisms to reduce their occurrence in effluents after a wastewater treatment plant [64,105]. Additionally, biodegradation seems to play the main role in pollutant removal [64,114], together with sunlight photodegradation and plant adsorption mechanisms [115]. The removal percentages of ARB and ARGs vary widely in CWs. For instance, Chen et al. (2016b) reported removal percentages between 75 and 98.6% for antibiotics such as erythromycin, monensin, clarithromycin, leucomycin, sulfamethoxazole, trimethoprim, sulfamethazine, and sulfapyridine [80,114]. Removal percentages between 63 and 84% were also reported for 12 ARGs, including three sulfonamide resistance genes (sul1, sul2, and sul3), four tetracycline resistance genes (tetG, tetM, tetO, and tetX), two macrolide resistance genes (ermB and ermC), two chloramphenicol resistance genes (cmlA and floR) in CWs operating with two different plant species in six flow configurations. Choi et al. (2016) reported a wider removal range for antibiotics, from 22 to 84%, depending on the antibiotic type. Sulfonamide-type antibiotics, with higher pKa values, are more effectively adsorbed into negatively charged soils; besides, sulfonamide antibiotics were more biologically degraded than other antibiotics in the presence of Phragmites australis and its associated microorganisms [115]. According to Huang X. et al. (2017), ARGs could be reduced by up to 97%, if CWs are operated in aerobic conditions for domestic effluents, because these effluents contain anaerobic intestinal microorganisms, which in turn can be retained in a CW support [113]. The CWs capable of removing ammonia nitrogen can also reduce ARGs in their effluents [64]. Therefore, CWs seems to be an appropriate technology for mitigating antimicrobial resistance. However, stabilizing a CWs operation can take a long time, up to three weeks [109], or inefficient when removing ARGs, if the wetlands operate at short retention times [116]. Other considerations are necessary to apply these systems to remove antibiotics to reduce the supports’ adsorption process. The long residence time of antibiotics or metals subjected to heterogeneous conditions within the CWs, along with the presence of other stressors, can also promote the microorganism’s antibiotic resistance by selective pressure [117,118]. Zhang et al. (2020) reported about the increase of sulfonamide resistance genes, as well as the increase of sulfamethoxazole concentration in the lower and the medium layers of the CWs [119]. On the contrary, Fang et al. (2017) analyzed the removal efficiencies of 14 ARGs (sul1, sul2, sul3, tetA, tetB, tetC, tetE, tetH, tetM, tetO, tetW, qnrB, qnrS, and qepA), intI1, and 16S rRNA genes in an integrated surface flow CW divided in four different subsystems [111]. The authors reported good ARG removal of up to 59.5% and 78% for summer and winter seasons, respectively, mainly due to removing total microbes in the treated water filtered by specific plant species such as *Phragmites australis*. However, the study also revealed a significant increase in the total concentration of nearly half of the genes detected in the final effluent in the subsystems operated at long term periods that promote sedimental microbial communities’ growth. The study also pointed out that mobile genetic elements or horizontal dissemination could also explain the observed increase of ARG in the winter, with low temperatures and low water flow velocities. Thus, further research is needed to figure out the optimal conditions and the mechanisms to achieve reproducible removal efficiencies of different types of ARGs in different influents of diverse quality.

### 2.3. Disinfection Treatments

Water disinfection is usually implemented at the end of the wastewater treatment process to reduce pathogenic microorganisms. Recently, disinfection using different techniques has gone from pathogenic inactivation to the destruction of genes, particularly those that confer bacterial resistance to antibiotics. Disinfection methods include chlorination, UV irradiation, and ozonation, being applied separately or as a combination of both [120,121]. These methods still offer a challenge to researchers in the field. Some publications have shown that ARB can be effectively removed under laboratory conditions by chlorination or UV [122], and up to 100% if chlorination and UV are combined as a treatment process [56,65]; moreover, the UV irradiation and low-level chlorine treatment reduced the gene transfer frequency by conjugation mechanisms [121], and ARGs genes can also be removed to different extents [56,74,121,123]. The reduction of ARGs (blaVIM, vanA, ampC, and ermB) ranged from 18.7% to up to 99.3% for ozonation. Meanwhile, ARB (Enterococci, Staphylococci, Enterobacteria, and *P. aeruginosa*) elimination varied from 60.2% to 98.9%. In another work, after ozone treatment, at the same time that the erythromycin resistance gene (ermB) was reduced by 2 orders of magnitude, vanA, blaVIM ARGs increased within the surviving wastewater population [124]. For these technologies, the type and size of the gene to be eliminated is an important variable. In the case of UV irradiation, it has been reported that the amount of adjacent T-T bases of the DNA fragment has a relevant role in its elimination. The higher the number of thymidines, the lower the degradation of the DNA fragment; whereas the elimination of ARB depends on the size of their genome and the number of adjacent cytidines, with an inversely proportional relationship between the variables, although directly proportional regarding the amount of thymidines [125]. Using UV irradiation and chlorination, the elimination of resistance genes that target tetracycline, ampicillin, sulfonamide, methicillin, and vancomycin in wastewater has been reported in effluents [74,125,126].

However, when these methods are applied in real wastewater conditions, the results showed that very low or insignificant ARB or ARG reduction and high UV fluence [83,120] or ozone doses and prolonged contact time are required [127]. It is even more concerning that some disinfection methods may contribute to select and to reconfigure microbial populations and their genes, shifting them towards resistance [124,128]. Consequently, these persistent bacteria can survive and spread resistance in the rest of the aqueous compartments, including the drinking water distribution system. Furthermore, the remaining DNA fragments can be acquired through the transformation mechanism. Therefore, it is not clear enough if disinfection technologies such as UV irradiation, chlorination, and ozonation are efficient in removing bacteria and resistance genes under existing conditions in WWTPs. Although disinfection technologies are well-established technologies, further studies should be conducted to overcome the multiple barriers found in the WWTP to eliminate ARB and ARG discharge into the environment [129].

### 2.4. Advanced Oxidation Processes

Advanced oxidation processes (AOP) are polishing technologies based on hydroxyl radicals, which have high oxidizing power and low selectivity, helpful in the oxidation and degradation of chemical substances to low or no toxicity compounds, as well as higher biodegradability [130,131]. Oxidative degradation includes Fenton oxidation, ozonation, and electrochemical and photocatalytic oxidation [132,133]. Some studies have shown that these methods remove ARB and ARGs to different extents and some antibiotics from real polluted water samples to a variable extent [74,122]. However, the effluent’s physicochemical conditions to be treated as pH and the amount of organic matter (biological and chemical) represent an important limitation to overcome [75,134]. These processes must generate enough hydroxyl radicals to degrade the biological and chemical matter present and carry out the oxidation of the intracellular material. Chen et al. (2020) reported that electrochemical oxidation removed ARB effectively, but nor for ARGs; on the other side, the electro-Fenton process was efficient for the removal of both intracellular and extracellular ARGs of ampC and tetA in wastewater from treatment plants and livestock production; however, the complete inactivation was not achieved [77]. The Fenton process involves a series of oxidation reactions to treat recalcitrant compounds or organic substrates in water matrices. The substrates are oxidized in the presence of ferrous ions or metal salts and hydrogen peroxide as the oxidizing agent by forming hydroxyl radicals. These radicals, which are strong oxidants, attack and destroy the organic pollutants at high reaction rates. The oxidation reaction can be improved by using ozone or UV-light to activate the H_2_O_2_ to form the hydroxyl radicals.

In another report, Moreira et al. (2018), using solar-H_2_O_2_, heterogeneous photocatalysis (with and without the addition of H_2_O_2_ and the photo-Fenton process, reported that the reduction on the ARGs was transient for intI1 and sul1 genes, and their abundance was observed to increase to original values following 3-day storage of the treated wastewater [135]. For the Fenton process, the optimal conditions for oxidation are usually different from current applications in WWTPs, such as acidic pH; when neutral pH is applied, the oxidation process is not efficient [75,136].

Photocatalysis is one of the most studied tertiary treatment technologies for the degradation of emerging pollutants; some industrial-scale examples exist, with titanium dioxide being the most used in the most recent studies. In these studies, the photocatalytic oxidation seems to be a promising technology for removing ARGs at the lab-scale [75,137], although some limitations have also been reported [128,138]. This technology presents similar limitations to the Fenton process. The operational pH must remain acidic, something not common in municipal or industrial effluents; furthermore, the applied light intensity optimization has yet to be studied for a better removal efficiency. The final destination of the catalyst, mostly if it is formed in nano-size, is still an issue to be solved as well as its operational stability [139]).

Undoubtedly, the trend of studies for the removal of bacteria and resistance genes points to hybrid technologies. Jiang et al. (2016) reported the fully inactivated antibiotic-resistance bacterium blaTEM-1 and aac(3)-II antibiotic-resistance genes via photoelectrocatalytic process, using semiconductor TiO_2_ nanotubes [138]. Ren et al. (2018) reported removing ARB and ARGs from a secondary wastewater effluent using a photocatalytic reactive membrane, using a polyvinylidene fluoride ultrafiltration membrane functionalized with nanoparticles of TiO_2_. The degradation efficiencies of the hybrid method were 97.82%, 20.66%, 99.45%, and 93.67% for floR, tetC, sul1, and intI1 in the plasmid [140]. Chen et al. (2020) reported that the electro-Fenton process was an effective method for removing ARB and both intracellular and extracellular ARGs [77]. In another report, Guo et al. applying UV/H_2_O_2_/TiO_2_ photocatalysis reported reduced intracellular and extracellular ARGs [137].

### 2.5. Nanomaterial-Based Treatments

With the development of nanotechnologies, new treatment methods against AMR include developing new drugs by the combination of antibiotics or pharmaceutical agents with metal nanoparticles or during the synthesis of antimicrobial polymers [16,141,142,143,144]. Antimicrobial polymers (AMP) were developed based on antimicrobial peptide properties to inhibit or kill bacteria [145]. Therefore, these materials can be used as antibiotics, disinfectants, and antiseptics in different applications [146]. These materials can be tailored to act as antibacterial agents by their intrinsic properties or functionalized with existing antibiotics to increase their antimicrobial efficiency [146]. Abouzeid et al. (2019) showed that nanocellulose materials are promising as a novel wastewater treatment, because they could be tailored as nanocrystals, nanofibrils, or nanofibers with enhanced adsorption capacities to remove heavy metals such as zinc, nickel, copper, cobalt, and cadmium, in a wide range of efficiency from 63% to 94% [147]. According to this study, a multilayered nanofibrous microfiltration system prepared with nanocrystals could retain bacteria such as *E. coli* and *B. diminuta*. It was highlighted that these materials have a high surface area, high capacity retention, they are environmentally inert, and they could have a high energetic value if burned, rendering them a promising alternative for wastewater treatment.

Particularly, nanomaterials have received considerable attention in the scientific community because of their great potential as a treatment technology in biomedical applications [148]. Among the microorganisms targeted by these new nanomaterials are the methicillin-, vancomycin-, and carbapenem-resistant pathogens, some strains from the ESKAPE bacteria virus. Some of these materials have proved to be effective in reducing the number of resistant bacteria (ARB) in wastewater, but less effective for removing ARG. Among the metals that have been used for nanoparticle synthesis and that have shown antimicrobial properties are iron, gold, palladium, and silver [141,144,149,150], with silver NPs (nanoparticles) being the most extensively used [143]. However, other rare metals, such as yttrium, could have potential antimicrobial properties that could represent a promising future for nanotechnology as a treatment process for antibacterial resistance (Rice, 2019). Metals present antimicrobial activity by depleting the levels of ATP of bacterial cells or inhibiting the respiratory enzymes, by damaging DNA or the protein and electron transport chain, or by generating reactive oxygen species that destroy the major molecular machinery of bacteria [141,149].

Nnaji et al. (2019) used nanobiocides to name nanomaterials with antimicrobial properties and classified them as metal oxide, carbon-based and natural nanomaterials. Thanks to their high antimicrobial activities, these materials are being used in medical treatments, air filters, and water treatment facilities [151]. For instance, silver nanoparticles alone or incorporated in biodegradable polymeric networks are the most researched materials that have been primarily used in water treatment processes. Other metals such as zinc, copper, and TiO_2_ have shown potential in inactivating Gram-positive bacteria, Gram-negative bacteria, and viruses. Apart from their high antimicrobial activity, several of these materials can be inexpensive, stable in water, and can be incorporated in thin films or nanotubes according to a specific application.

Like functionalized polymers, activated carbon nanoparticles derived from biowaste have been proven to present appropriate antimicrobial or antifungal activities [152,153] that could be useful in water purification processes. Mainly, nanoparticles adhere to the microorganism cell to modify the membrane charges causing cell disruption, destroy the microorganism DNA, or prevent bacteria from the cell division leading to cell death. When synthesized into carbon nanoparticles, the principal advantages of these materials are that they can be synthesized to diameter sizes as small as 2 nm and up to 48 nm. Composites with silver nanoparticles could be obtained with higher antibiotic activity; pathogenic and non-pathogenic bacteria such as *E. coli*, genus *Bacillus* *S. aureus*, *K. pneumoniae*, *C. violaceum*, *P. notatum*, *P. aeruginosa*, and the fungus *Candida albicans* could be well destroyed or inhibited. Nanoparticles from coconut or sugarcane bagasse report high surface areas (up to 1489 m^2^.g^−1^), and most of the carbon nanoparticles need not be activated with aggressive chemicals, reducing the environmental impact. The authors conclude that these nanoparticle synthesis presents a high potential as antimicrobial agents, because of their low-cost production and their high specificity and efficiency [152].

Despite all the antimicrobial properties of nanoparticles, these can also present toxic properties to mammalian cells or promote antibiotic resistance and ARGs. NPs have been used in the antibacterial assessment of pathogenic bacteria [154]. However, bacteria could also develop metal resistance by different mechanisms, such as biofilm production or the proliferation of metal resistant genes [143]. In other words, bacteria or pathogens may be destroyed, but their genes could still be transferred to other bacteria or microorganisms, promoting microbial resistance.

## 3. Conclusions

One of the determinants in the problem associated with microbial resistance is the technological capacity of elimination. The WWTPs are currently the physical areas where the elimination of persistent and emerging compounds occurs with acceptable removal rates. In the short and medium-term, WWTP should be updated with technological innovations to facilitate the elimination of ARB and ARGs and other emerging pollutants. However, these technologies must be equally efficient in removing ARB and ARGs to mitigate the environmental and human health impact of microbial resistance. To date, many studies on the different technologies have been carried out to study their elimination capacity, to optimize their efficiency, to understand the mechanisms of action of the different technologies, to elucidate the limitations found in physicochemical conditions in WWTPs, and also to know and overcome the reasons why WWTPs are sites of amplification and selection of ARB. For the prevention and control of microbial resistance, mitigation treatments must necessarily be accompanied by procedures for continuous monitoring of the prevalence of ARB in environmental compartments, updating or creating regulations for hospital effluents, in the proper management of excreta from units of animal production, and in programs to control the use of antibiotics in livestock and the agricultural industries. In addition, stringent policies and programs to control medical drug consumption and management of human antibiotic waste are required.

## Figures and Tables

**Table 1:** Examples of microorganisms found in different wastewater treatment plants.

Microorganism or Resistant Strain	Resistance Profile	Water Sample	Country or Place	Reference
*Pseudomonas* *Enterococcus*	Penicillin G, Ampicillin, Vancomycin, Erythromycin, Triple sulfa, and Trimethoprim /sulfamethoxazole	Influent and effluent from wastewater treatment plant (WWTPs)	Germany	[42]
*Acinetobacter* spp	Trimethoprim, rifampin, chloramphenicol	Influent, effluent wastewater treatment plant and receiving body of the plant (River)	Michigan, USA.	[43]
*Escherichia coli* *Enterococcus faecium*, *Enterococcusfaecalis*	Ampicillin, Tetracycline, Erythromycin	Influent and effluent, as well as in the aeration chamber and in the return activated sludge mixture	Poland	[44]
*Staphylococcus aureus* methicillin-resistant (MRSA)	Multi-resistant	Affluent treatment plant	USA	[45]
*Enterococcus* vancomycin-resistant (ERV)	Multi-resistant	Non-chlorinated effluent	USA	[46]
Resistance genes	Sulfonamide(sul), Macrolides(erm), Tetracycline(tet) and Quinolones (qnr)	Crude affluent, Primary clarifier tank, Anaerobic tank. Aerated tank, secondary clarifier Final effluent	China	[47]
Bacterial isolates resistant to tetracycline (*Escherichia y Serratia*)	Multi-resistant	Wastewater from the secondary treatment process of three WWTPs	Toronto	[48]
Enterococos *E. faecalisy* *E. faecium*	Multi-resistant	Primary effluent, final effluent, and biomass.	Canada	[49]
*Escherichia coli*, *Klebsiella* spp, ^Aeromonas^ spp.	Ciprofloxacin, Cotrimoxazole, Ampicillin, and Trimethoprim	Affluent and Effluent from the WWTP	City of Sneek, The Netherlands	[41]
*Escherichia coli*	Ampicillin, Cefazolin, and Ceftriaxone	Sludge from a WWTP	Taizhou, China	[50]
*Pseudomonas*, *Staphylococcus*, Streptococcus	Multi-resistant	Affluent and effluent	Florida	[51]
*Escherichia coli*	Amoxicillin, ciprofloxacin, norfloxacin, kanamycin, sulfamethoxazole/trimethoprimand tetracycline	Sludge in the aeration tank and return sludge	Japan	[52]
Resistance genes	Sulfonamides (sul1), tetracycline (tetM) and polymixin (mcr-1) and of the class 1 integrase gene (intI1)	16 different European effluents of WWTPs	Europe	[53]
Resistance genes	Tetracycline (tet A, B, C, G, L, M, O, Q, X) and sulfonamide (sulI, sulII, sulIII)	Raw influent and final effluent samples	Poland	[54]
Resistance genes	Chloramphenicol (catA1); sulfonamides (sul I); tetracycline (tetE); aminoglycoside (aac (3)) -IV; penicillins bla _TEM_, bla _CTX-M_, bla _NDM-1_	Pharmaceutical wastewaters	Nigeria	[55]
Resistance genes	Sulfonamides (sul1, sul2), tetracycline (tetW, tetQ, tetX)	Activated sludge	China	[56]
The extended-spectrum beta-lactamase (ESBL)-producing *Escherichia coli*	Ampicillin, cefazolin, and ceftriaxone	Aerobic active sludge	Taizhou, China	[50]
Resistance genes	Sulfonamides (sul1, sul2) Tetracyclines (tetO, tetQ, tetW)	Effluent of secondary treatment in WWTPs	Europe, America, Asia, and Africa	[57]
*Salmonellar*	Tetracycline, Streptomycin, kanamycin,	Sewage influent of WWTPs	Japan	[58]
*Campylobacter*, *Salmonellar* spp., *Escherichia coli* *O157*	Ciprofloxacin, nalidixic acid erythromycin, Streptomycin, gentamicin	Influents and effluents from WWTPs	Italy	[59]
Resistance genes	Tetracyclines (tetA, tetB, tetM, tetX), beta-lactams (bla_TEM_, bla_SHV_, bla_OXA_, bla_CTX-M_, bla_CTX-M-1_, bla_CTX-2_, bla_CTX-M-9_, bla_VEB_, bla_CMY_, bla_AMP-C_), chloramphenicol (florR, cmlA, fexA, fexB)	Samples of untreated wastewater and treated wastewater from 4 WWTPs	Poland	[60]
*Staphylococcus* spp.	Methicillin, vancomycin	Activated sludge bioreactor	Olsztyn, Poland	[61]

**Table 2:** Categorization of treatment processes (adapted from references [67,68,69]).

Category	Pathway or Mechanisms	Advantages/Drawbacks
Conventional wastewater treatment processes	A combination of physical (settling ponds), chemical (coagulation/flocculation), and biological processes (aerobic/anaerobic)	Acclimation of micro sludge fauna can lead to carrying antibiotic resistance to the environment
Tertiary and advanced treatment processes	Advanced separation techniques (membrane filtration, distillation, reverse osmosis, adsorption on activated carbon)	Membrane filtration or adsorption represents a transfer/concentration of pollutants to a matrix that is disposed of as solid residues
Advanced oxidation processes	Ozonation, Fenton oxidation, photocatalysis, plasma technology, ultrasonic technology	Good efficiency of antibiotic degradation/ Can generate unknown byproducts or more toxic than parent compounds
Hybrid treatments (combination of technologies)	Membrane bioreactors or use of synthetic biology such as enzymatic removal of active pharmaceutical ingredients	Good efficiency of antibiotic degradation/ Generation of unknown byproducts with an enzymatic process
Post conventional treatment processes	Constructed wetlands	Represents the concentration of antibiotics in soil or plant roots. Further studies on biodegradation mechanisms are needed.

**Table 3:** Examples of technologies treatment for antimicrobial resistance mitigation.

Microorganism or Resistant Strain	Operating Conditions	Treatment	Country or Place	Reference
Resistance genes (tet (O), tet (W), sulI, sulII)	Test thermophilic digesters were amended with environmentally relevant concentrations of Ag NP (0.01, 0.1, and 1.0 mg-Ag/L	Thermophilic anaerobic digesters	Virginia, USA	[70]
Resistance genes (tet (A), tet (L), tet (O), tet (W), and tet (X)) and the gene encoding the integrase (intI1) of class 1 integrons	The anaerobic reactors at 37 °C, 46 °C, and 55 °C	Anaerobic reactors	Minnesota, USA	[71]
*Staphylococcus aureus*, *Escherichia coliyKlebsiella pneumoniae*	Reaction time for disinfection is 180–240 and 90–120 min, respectively	Solar light and solar photo-Fenton processes	Switzerland	[72]
Resistance genes (tetA y bla _TEM-1_)	Photo-Fenton under visible LED and neutral pH conditions.	Photo-Fenton	Australia	[73]
Resistance genes (sul1 y tetG)	Dose of 160 mg/L with a contact time of 120 min	Chlorination	China	[74]
Resistance genes (sul1, tetX y tetG)	pH was 3.5 with an H_2_O_2_ concentration of 0.01mol/L accompanied by 30min of UV irradiation	UV/H_2_O_2_ process	China	[75]
*Escherichia coli*	H_2_O_2_/TiO_2_/sunlight (cumulative energy per unit of volume (QUV) in the range 3–5 Kj/L	Disinfection and solar-driven advanced oxidation processes	Italy	[76]
*Escherichia coli* and *P. aeruginosa*	Current density from 7.14 mA/cm^2^ to 21.42 mA/cm^2^ and 120 min of treatment	Electrochemical	China	[77]
*Escherichia coli* NDM-1	Bi_2_O_2_CO_3_ microspheres wrapped with nitrogen-doped reduced graphene oxide (NRGO)	Photocatalytic process	China	[78]
*Escherichia coli*	Ultrafiltration (UF) and nanofiltration (NF) membranes	Nano- and ultra-filtration processes	Norway	[79]
*Mycobacterium*, *Ferruginibacter*, *Thermomonas*, *Morganella*, *Enterococcus*, *Bacteroides*, *Myroides y Romboutsia*	UV dosage the 320 mJ/cm^2^ and dose chlorine 1–2 mg/L	Combined UV and chlorine process	China	[57]
Resistance genes sul1, sul2 and sul3,tetG, tetM, tetO tetX, ermB, ermC, cmlA and floR	Surface flow, horizontal subsurface flow, and vertical subsurface flow and two Plant species (Thaliadealbata Fraser and Iris tectorum Maxim)	Constructed wetlands (CWs)	China	[80]
*Escherichia coli*	High current pulsed irradiation of 280 nm LEDs	Pulsed UV-LED irradiation	China	[81]
Resistance genes Sul1	UV dose 432 mJ/cm2 and chlorine dosage 10 mg/L for small fragments and 40 mg/L for large fragments	Combined UV/free chlorine processes	Singapore	[82]
*Escherichia coli* *y* *Enterococcus faecium*	1 mg/L of ozone, with a contact time of 5 min	Ozone treatment	Germany	[83]
*Escherichia coli*	Silver decorated graphene oxide (Ag/GO) composite and 60 min illumination	Nanomaterial-based treatments	China	[84]
Resistance genes tetA, tetC, msrSA y ermB	Ventilated sludge drying reed bed	Wetlands	China	[85]
Resistance genes bla _TEM_, ermF, mecA y tetA	Free chlorine dosage of 30 mg/L with a 30-min contact time	Chlorination	Louisiana, USA	[86]

## Abbreviations

ARB	Antibiotic-resistant bacteria
ARG	Antibiotic resistance genes
AMR	Antimicrobial resistance
AOP	Advanced oxidation processes
AMP	Antimicrobial polymers
BOD	Biological oxygen demand
COD	Chemical oxygen demand
CWs	Constructed wetlands
NF	Nanofiltration
NP	Nanoparticles
MBR	Membrane bioreactor
UF	Ultrafiltration
